# Superionic Al─O─H Compounds With Unconventional Stoichiometries Under Extreme Planetary Conditions

**DOI:** 10.1002/advs.78561

**Published:** 2026-11-10

**Authors:** Zifan Wang, Ji Hoon Shim, Duck Young Kim

**Affiliations:** ^1^ Center for High Pressure Science & Technology Advanced Research (HPSTAR) Shanghai P. R. China; ^2^ Department of Chemistry Pohang University of Science and Technology Pohang Republic of Korea; ^3^ Division of Advanced Nuclear Engineering Pohang University of Science and Technology Pohang Republic of Korea

**Keywords:** aluminum oxyhydrides, high‐pressure chemistry, planetary interiors, structure prediction, superionic conductors

## Abstract

Extreme pressure and temperature induce novel chemical behaviors, including unconventional stoichiometries and superionic states. Here, by combining crystal structure prediction with first‐principles calculations, we identify two unconventional stoichiometries in the ternary Al─O─H system, AlOH and AlH_3_O_4_, which lie on the convex hull at 300 GPa. Representative formation reactions become thermodynamically stable above 65 and 245 GPa after including ZPE, respectively. Both compounds are wide‐gap insulators and AlH_3_O_4_ exhibits pronounced site‐dependent charge differentiation between inequivalent oxygen environments under high pressure. Crucially, ab initio molecular dynamics simulations reveal protonic superionic transitions at elevated temperatures, where protons diffuse like a liquid through rigid Al─O sublattices. High‐pressure and high‐temperature dynamical‐state diagrams further indicate that the solid, superionic, and liquid regimes overlap with conditions expected in deep planetary interiors, suggesting that they could play important roles in chemical behaviors inside planetary bodies. These findings significantly enrich the high‐pressure phase diagram of Al─O─H system and provide valuable insights into chemical behaviors under extreme conditions.

## Graphical abstract

First‐principles calculations reveal stable AlOH and AlH_3_O_4_ phases and protonic superionic behavior at high pressures and temperatures. Hydrogen diffuses rapidly through crystalline Al─O frameworks, while the two compounds exhibit different superionic transition trends with pressure. These results broaden high‐pressure Al–O–H chemistry and suggest efficient proton transport under extreme planetary conditions.

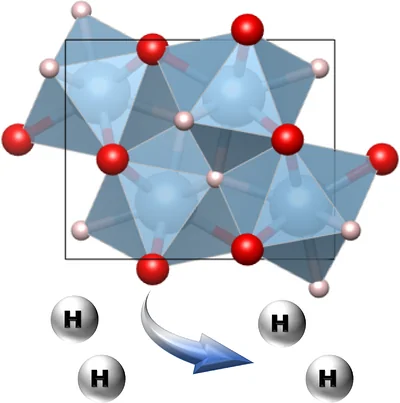

## 1 Introduction

Pressure serves as an effective thermodynamic dimension that forces elements to challenge conventional chemical rules, leading to the emergence of unexpected chemical stoichiometries [1, 2, 3, 4]. At ambient conditions, atoms in compounds are strictly governed by fixed electronic configurations and valence rules. However, under extreme compression, these traditional constraints are often violated, giving rise to novel compounds with unexpected compositions. For instance, sodium chloride deviates from the conventional 1:1 stoichiometry under high pressure, forming numerous unconventional stoichiometries such as Na_3_Cl and NaCl_3_ [2]. Similarly, unexpected iron peroxide FeO_2_ was discovered at extreme pressure relevant to Earth's interior, providing a new geochemistry perspective on the deep‐Earth oxygen cycle [3].

Beyond pressure, temperature plays a pivotal role in inducing exotic states of matter, most notably the superionic state. Superionic state is a unique state where materials exhibit liquid‐like mobility within a solid crystal lattice. Under ambient conditions, this property is essential for designing solid electrolytes for solid‐state batteries [5, 6]. At extreme pressures and temperatures, solid phases can transform into superionic states, which is found to occur in a wide variety of deep‐Earth minerals [7, 8, 9], as well as icy compounds within planetary interiors such as Uranus and Neptune [10, 11, 12, 13, 14].

The ternary Al─O─H system represents a promising frontier for exploring such exotic chemical behaviors under extreme planetary conditions. Currently, our understanding of this system is dominated by the chemically stoichiometric compound AlOOH and its high‐pressure polymorph: δ‐AlOOH, which serve as a major water carrier in Earth's mantle [15, 16]. δ‐AlOOH is a high‐pressure phase of α‐AlOOH (diaspore) and γ‐AlOOH (boehmite), which is identified to be stable above 17 GPa up to at least 134 GPa and approximately 2000 K [17]. Extensive studies have mapped its structural evolution under high pressure, including the transitions to a pyrite‐type phase above 170 GPa [18], a monoclinic *P21/c* phase at 340 GPa [19], an orthorhombic *Pbca* phase at 160 GPa [20], and an orthorhombic ε‐phase at ∼190 GPa [21]. Beyond these structural transitions, the dynamic behavior of δ‐AlOOH under extreme conditions has also attracted significant attention. Recent computational simulations have revealed that δ‐AlOOH transitions into a superionic state under deep‐mantle conditions, where protons exhibit liquid‐like diffusion within a rigid Al‐O framework [22, 23]. Particularly, Luo et al. employed machine‐learning potentials to probe the microscopic dynamics of proton diffusion in δ‐AlOOH [22], while He et al. further revealed a double superionic transition that effectively stabilizes the phase against dehydration near Earth's core‐mantle boundary [23]. However, despite the findings on AlOOH, the chemical landscape of Al─O─H system beyond this conventional stoichiometry remains largely unexplored. Specifically, it remains an open question whether the ternary system allows for the stabilization of other thermodynamically stable phases with unconventional stoichiometries, and if these phases can exist and exhibit superionic behaviors under more extreme planetary conditions.

In this study, by employing systematic crystal structure prediction combined with first‐principles calculations, we extensively explore the Al─O─H compositional space. We identify two unconventional stoichiometries, AlOH and AlH_3_O_4_, that lie on the ternary convex hull at 300 GPa. Representative formation‐reaction calculations further show ZPE‐corrected crossover pressures of approximately 65 and 245 GPa, respectively. Furthermore, ab initio molecular dynamics (AIMD) simulations reveal that the two compounds undergo superionic transitions at elevated temperatures. These results expand the known high‐pressure chemistry of the Al─O─H system and provide a basis for exploring its dynamical behavior under extreme planetary conditions.

## 2 Results and Discussion

By performing systematic crystal structure predictions, we explored the potential energy surface of ternary Al─O─H system at 100 and 300 GPa, which reveals a chemical landscape far richer than previously assumed. The reliability of our search result is validated by the successful reproduction of established binary phases in the H─O, Al─O, and Al─H systems [24, 25, 26, 27, 28, 29, 30], as shown in the convex hull diagram (Figures S1–S3). Most significantly, within the ternary region, we identified two novel thermodynamically stable compounds: *Pnma*‐AlOH and *P‐1*‐AlH_3_O_4_ (Figure 1a,c). AlOH lies on the convex hull at both pressures, whereas AlH_3_O_4_ is stabilized on the convex hull at 300 GPa. While AlOH represents a ternary extension of simple hydroxides, AlH_3_O_4_ distinguishes itself as a hydrous phase containing an exceptional water content of 18.6 wt.%, which far exceeds that of many conventional mantle minerals. Detailed structural parameters for these predicted phases are provided in Table S1.

**FIGURE 1:**
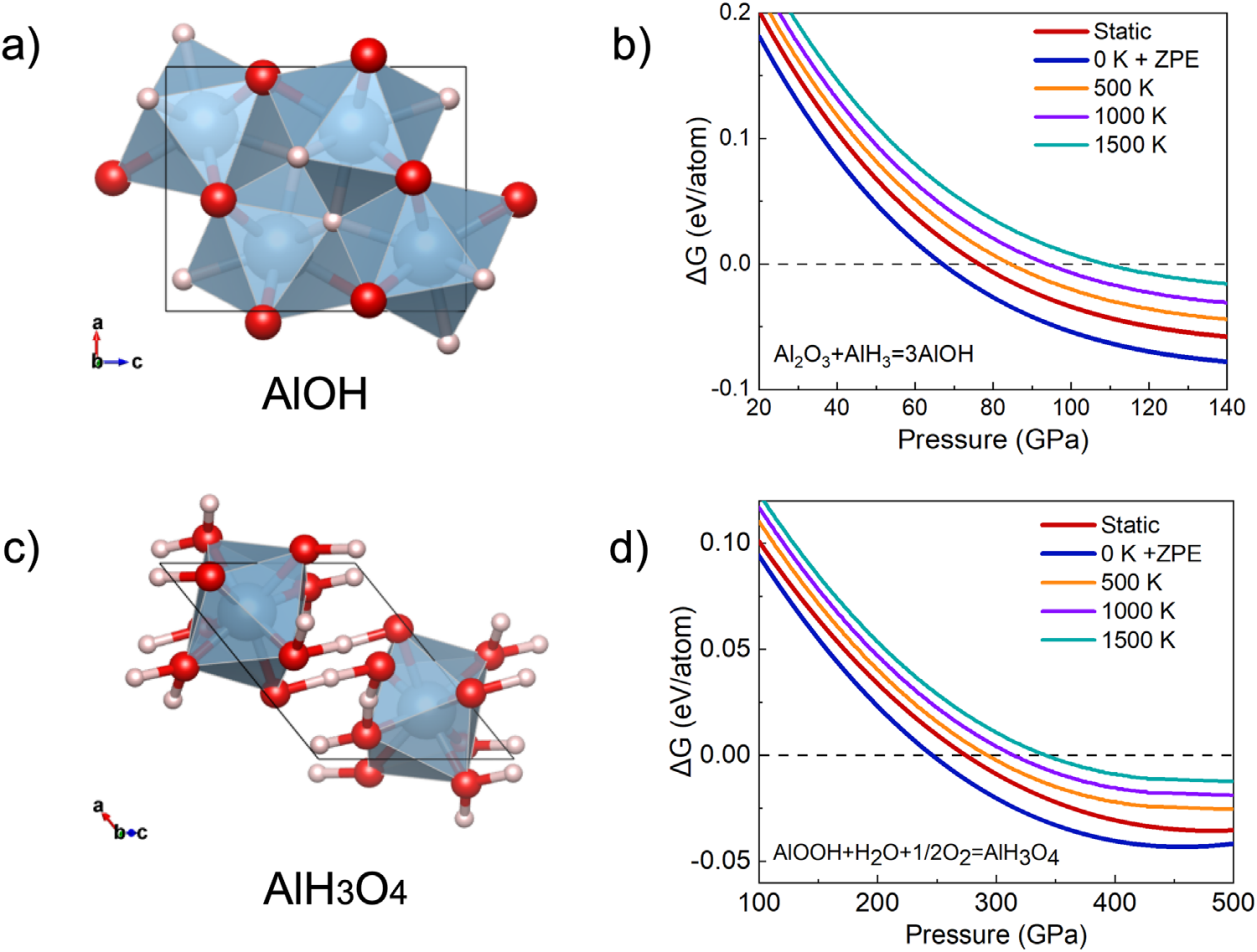
Crystal structures and formation enthalpy of AlOH and AlH_3_O_4_.(a, c)The crystal structure of AlOH(a)and AlH_3_O_4_(c)at 300 GPa. The blue, white and red spheres correspond to Al, H, and O atoms, respectively.(b)Pressure dependence of the reaction free‐energy change for Al_2_O_3_ + AlH_3_ → 3AlOH.(d)Pressure dependence of the reaction free‐energy change for AlOOH + H_2_O + 1/2O_2_ → AlH_3_O_4_. The static enthalpy, the 0 K result including zero‐point energy(ZPE), and the Gibbs free‐energy changes at 500, 1000, and 1500 K are shown for comparison.

To further evaluate the pressure and temperature‐dependent reaction energetics of these phases, we considered two representative formation reactions: Al_2_O_3_ + AlH_3_ → 3AlOH and AlOOH + H_2_O + 1/2O_2_ → AlH_3_O_4_. At 0 K, the static reaction enthalpies become negative above approximately 75 and 275 GPa for AlOH and AlH_3_O_4_, respectively, while inclusion of zero‐point energy lowers the corresponding crossover pressures to approximately 65 and 245 GPa (Figure 1b,d). We further included vibrational free‐energy contributions to evaluate the finite‐temperature Gibbs free energy changes of these reactions. Increasing temperature systematically shifts the crossover pressures to higher values, reaching approximately 105 GPa for AlOH and 340 GPa for AlH_3_O_4_ at 1500 K. These results demonstrate that vibrational entropy has a significant effect on the reaction energetics under planetary temperatures. These crossover pressures describe the energetics of the specified reactions and should not be interpreted as global thermodynamic stability boundaries.

In addition, we also examined the direct deoxygenation pathway AlOOH → AlOH + 1/2O_2_ (Figure S4). This reaction remains energetically unfavorable over the investigated pressure range, indicating that AlOH should not be regarded as a simple deoxygenation product of δ‐AlOOH under the conditions considered. Compared with δ‐AlOOH, which is relevant to hydrogen transport in the deep mantle, AlH_3_O_4_ represents a substantially H‐richer Al─O─H stoichiometry. However, the pressure range relevant to the formation of AlH_3_O_4_ in our calculations lies above those in Earth's mantle, indicating that it is unlikely to play a direct role in the present‐day terrestrial deep‐water cycle. Instead, AlH_3_O_4_ may represent an extreme‐pressure extension of Al─O─H chemistry that could become relevant in the deeper interiors of larger rocky planets [31, 32, 33, 34].

Beyond thermodynamic stability, we further verified the dynamical stability of these compounds via phonon dispersion calculations. The calculated phonon spectra demonstrate that AlOH and AlH_3_O_4_ remain dynamically stable over a wide pressure range: AlOH is dynamically stable from 10 GPa to at least 1 TPa, AlH_3_O_4_ exhibits remarkable stability starting from ambient pressure up to at least 1 TPa. They exhibit no imaginary frequencies over the investigated pressure ranges, as shown in Figures S5 and S6 in the SM.

To evaluate the electronic evolution under compression, we calculated the band structures of AlOH and AlH_3_O_4_ across their dynamical stability range. The band structures of AlOH and AlH_3_O_4_ at 300 GPa are illustrated in Figure 2a,b, and the detailed band structures are summarized in Figures S7 and S8 in the SM, indicating that both compounds are insulators with wide band gaps through their stable pressure ranges. Generally, extreme compression is expected to close the band gap of solids and drive them toward metallization, particularly in hydrogen‐rich systems [35, 36, 37]. However, instead of narrowing, the calculated PBE Kohn–Sham gaps of both compounds increase with pressure, as shown in Figure 2c. Specifically, the band gap of AlH_3_O_4_ expands from 4.15 eV at 10 GPa to 5.65 eV at 1 TPa, and the band gap of AlOH increases from 3.05 eV at 10 GPa up to 7.24 eV at 1 TPa. We note that PBE generally underestimates absolute band gaps, and the calculated values should not be interpreted as quantitatively accurate quasiparticle gaps. Nevertheless, the calculations consistently reveal a pressure‐induced gap‐widening trend within the same theoretical framework.

**FIGURE 2:**
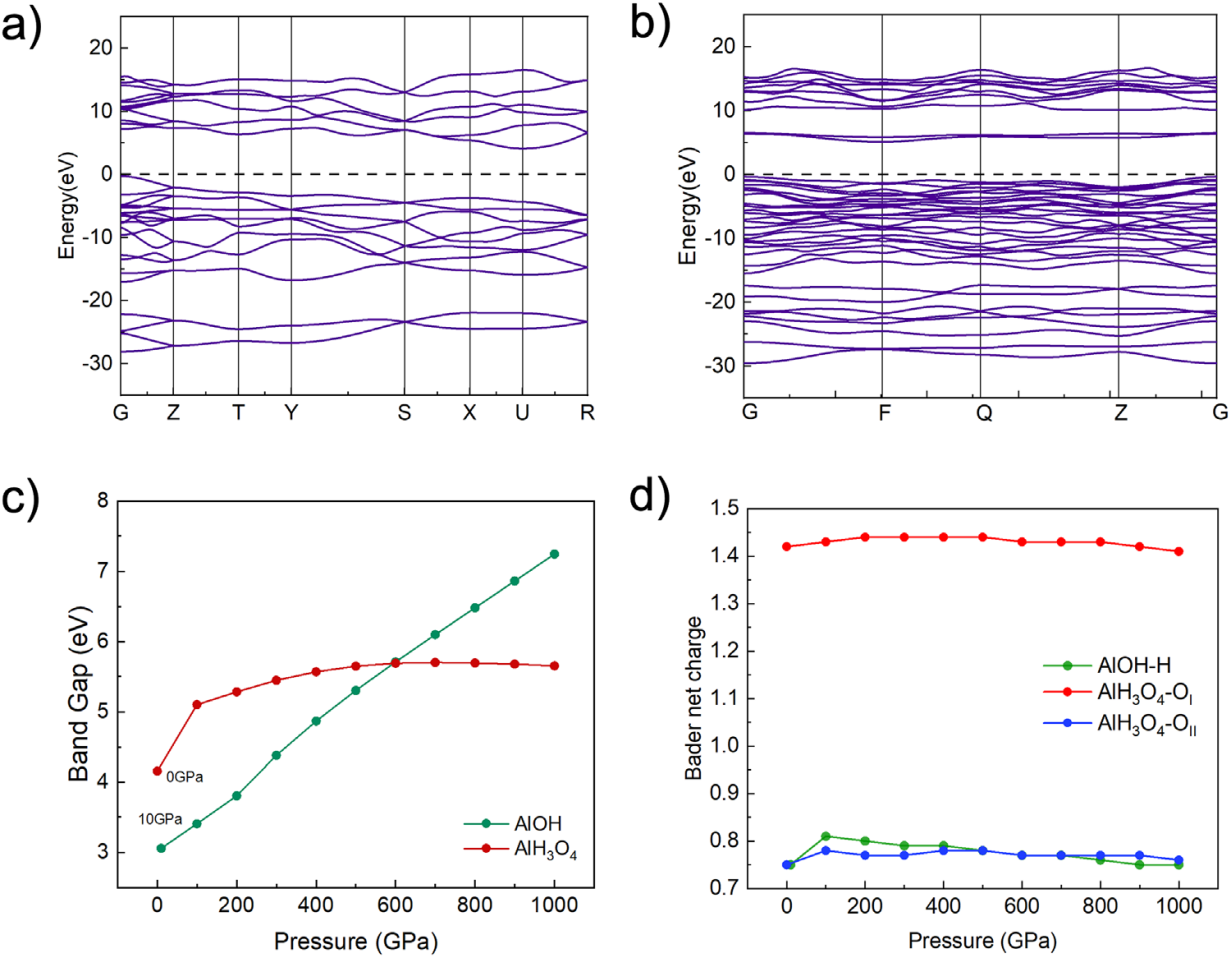
Electronic structures of AlOH and AlH_3_O_4_. (a, b) Band structures of AlOH (a) and AlH_3_O_4_ (b) at 300 GPa. (c) Pressure dependence of band gaps of AlOH under 10–1000 GPa and AlH_3_O_4_ under 0–1000 GPa. (d) Pressure dependence of bader net charges of AlOH and AlH_3_O_4_ under 0–1000 GPa.

To elucidate the chemical origin of these unconventional stoichiometries, we analyzed their charge distribution and local electronic structures. The Bader charges remain relatively insensitive to pressure over the investigated range (Figure 2d). In AlOH, H exhibits a negative Bader charge of approximately −0.77 *e*, indicating an electron‐rich, hydride‐like environment rather than the protonic H characteristic of conventional hydroxides. Together with the Bader charges of approximately +2.44 e for Al and −1.65 e for O, this charge distribution accounts for the charge neutrality of the unconventional AlOH stoichiometry. In AlH_3_O_4_, two inequivalent oxygen groups exhibit distinctly different Bader charges: O_I_ (O1–O4) carries approximately −1.43 e, whereas O_II_ (O5–O8) carries approximately −0.77 e. These Bader charges are used here as descriptors of charge distribution rather than formal oxidation states.

Accordingly, the two oxygen groups in AlH_3_O_4_ exhibit markedly different local coordination environments. At 300 GPa, O_I_ sites show short O─H distances of 1.024–1.042 Å and nearest O─O distances of 2.061–2.067 Å, whereas O_II_ sites exhibit O─H distances of 1.097–1.126 Å together with much shorter O–O contacts of 1.309 Å. ELF analysis further reveals distinct local electron‐localization patterns for these environments (Figure S9). In AlOH, pronounced electron localization is observed around H, consistent with its electron‐rich character. The site‐resolved PDOS further shows clearly different O‐2p spectral distributions for O_I_ and O_II_ in AlH_3_O_4_ over the investigated pressure range (Figure S10), confirming that the two oxygen groups possess distinct local electronic environments. These results indicate pronounced site‐dependent charge differentiation associated with distinct local bonding environments.

We further examined the origin of the pressure‐induced band gap widening by analyzing the pressure‐dependent PDOS (Figure S10). In AlOH, the valence‐band‐edge states are dominated by O‐2p orbitals with appreciable H‐1s hybridization, whereas the low‐lying conduction states are mainly Al‐s, p derived. In AlH_3_O_4_, substantial O‐2p character occurs at both band edges, with O_I_ and O_II_ exhibiting distinct pressure‐dependent spectral distributions. The relatively weak H‐1s contribution at the band edges indicates that the gap widening is associated with pressure‐induced changes in orbital hybridization within the Al─O─H framework.

To investigate their dynamical behavior under extreme conditions, we performed extensive AIMD simulations. From AIMD simulations, we calculated the mean squared displacements (MSD) of different atoms and atomic trajectories within the lattice structures of AlOH and AlH_3_O_4_, as respectively shown in Figure 3. At relatively low temperatures, the MSDs of all species remain small and approximately constant close to zero, indicating that all the atoms vibrate and are confined near their equilibrium positions (Figure 3a,d,g,j). Under this condition, the diffusion coefficients of *D_H_* = *D_O_* = *D_Al_* = 0, signaling that the structures are in conventional solid states. As temperatures increase, the MSD of hydrogen in AlOH and AlH_3_O_4_ exhibit approximately linear long‐time growth, yielding substantial diffusion coefficients of *D_H_* = 1.47 × 10^−8^ m^2^ s^−1^ at 400 GPa and 7000 K for AlOH and *D_H_* = 2.09 × 10^−8^ m^2^ s^−1^ at 330 GPa and 3000 K for AlH_3_O_4_, while the others remain localized around their equilibrium positions with *D_O_* = *D_Al_* = 0, suggesting the transitions into superionic states at the temperatures (Figures 3b,e,h,k). The hydrogen sublattice melts in superionic states, exhibiting liquid‐like diffusion through the solid Al─O framework. At higher temperatures, all atomic species diffuse freely, *D_H_*, *D_O_*, and *D_Al_* are all nozero, implying the melting of the structures and the transitions into liquid phases (Figure 3c,f,i,l). The robustness of the assigned superionic states was further verified by extended 30 ps AIMD simulations and 200 ps large‐scale machine learning molecular dynamics (MLMD) simulations containing 1152 atoms for AlOH and 1024 atoms for AlH_3_O_4_ (Figures S13 and S14). Unlike δ‐AlOOH, AlH_3_O_4_ shows no distinct double‐superionic regime within the investigated conditions; H diffusion precedes melting, while appreciable Al and O diffusion appears only in the liquid state.

**FIGURE 3:**
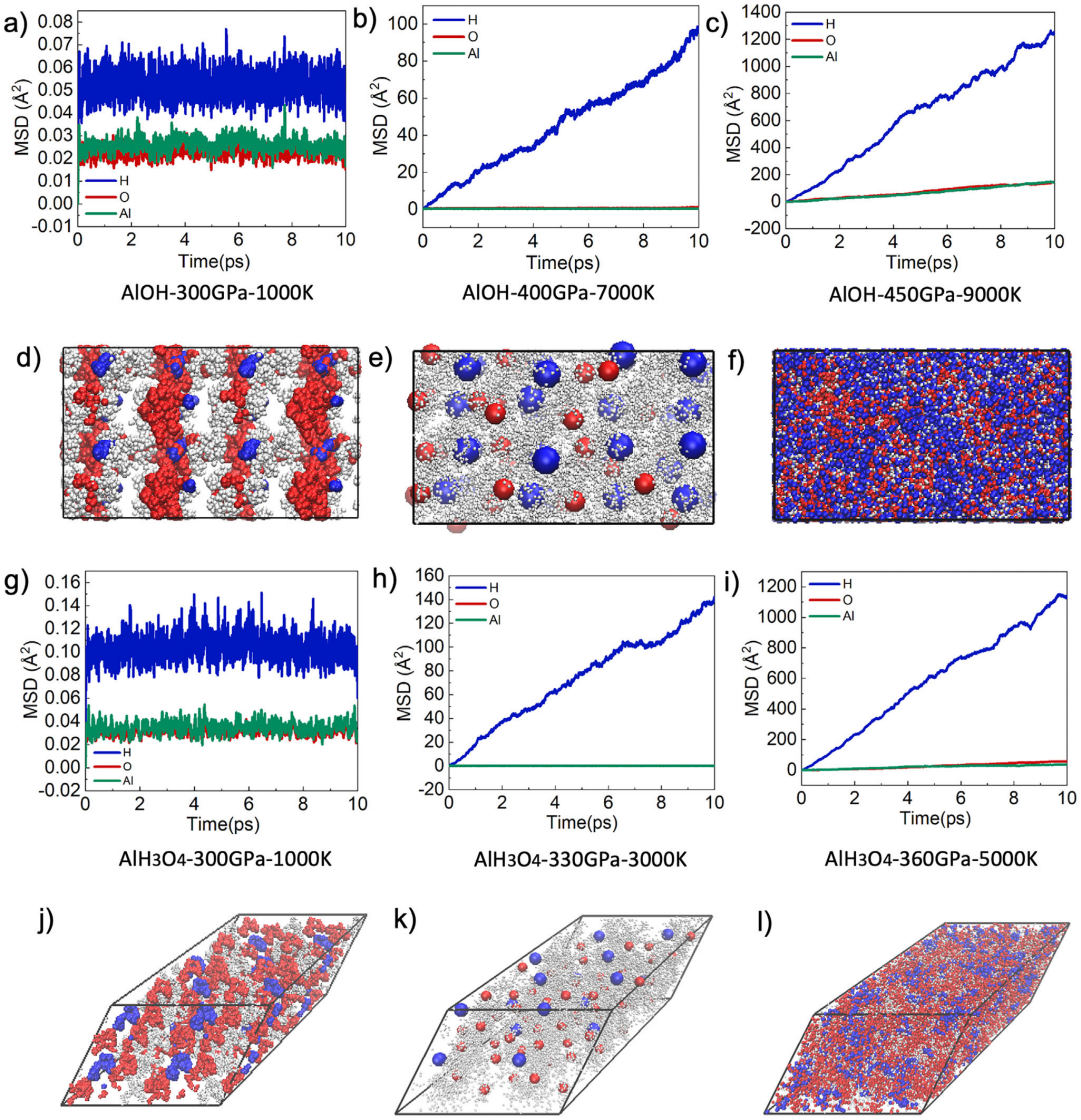
Dynamical behaviors of AlOH and AlH_3_O_4_ at extreme conditions. (a–f) Calculated mean squared displacement (MSD; upper panel) and atomic trajectories (lower panel) of AlOH structure at: (a) and (d) 300 GPa and 1000 K, (b) and (e) 400 GPa and 7000 K, (c) and (f) 450 GPa and 9000 K. (g–l) Calculated mean squared displacement (MSD; upper panel) and atomic trajectories (lower panel) of AlH_3_O_4_ structure at: (g) and (j) 300 GPa and 1000 K, (h) and (k) 330 GPa and 3000 K, (i) and (l) 360 GPa and 5000 K.

Based on the AIMD simulations, we constructed the high‐pressure and high‐temperature dynamical‐state diagrams of AlOH and AlH_3_O_4_ (Figure 4). Three distinct dynamical regimes are identified for AlOH and AlH_3_O_4_: a rigid solid, a diffusive superionic phase, and a fully disordered liquid. For AlOH, the solid phase is remarkably robust, requiring high temperatures to trigger the superionic transition. Specifically, the transition temperatures are ∼3800 K at 140 GPa and ∼6500 K at 980 GPa for AlOH. In contrast, the solid–superionic crossover in AlH_3_O_4_ shows only a weak positive pressure dependence and remains substantially less sensitive to compression than that of AlOH. The weaker pressure sensitivity of AlH_3_O_4_ is consistent with its more heterogeneous local bonding environment, which provides a broader range of configurations accessible to mobile H, making proton transport less sensitive to compression. Consequently, AlH_3_O_4_ possesses a wider superionic range than AlOH, retaining the diffusive protonic properties up to melting temperatures of ∼4500 K.

**FIGURE 4:**
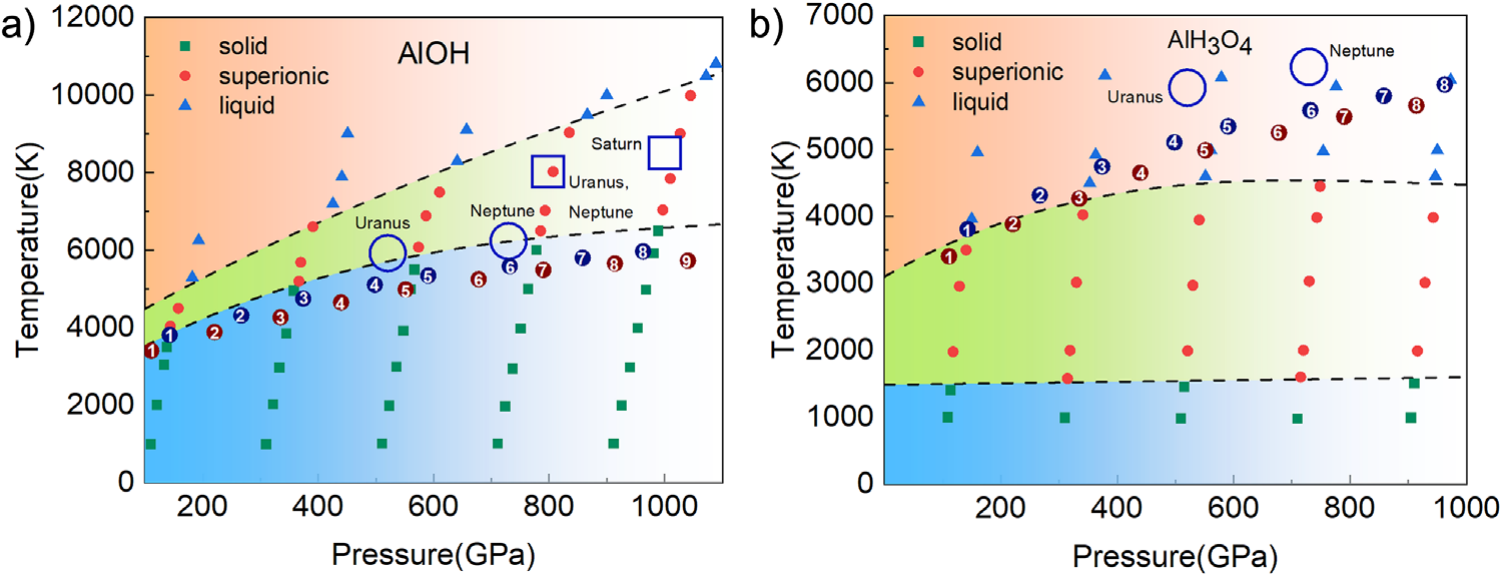
Pressure–temperature dynamical‐state diagrams for AlOH (a) and AlH_3_O_4_ (b). Blue, green, and orange regions represent the solid, superionic, and liquid regions, respectively; green squares, red circles, and blue triangles represent solid, superionic, and liquid states, respectively. The brown and blue circles display the pressure‐temperature conditions at the core mantle boundary (CMB) of terrestrial and ocean type exoplanets, respectively, while numbers in those circles represent the planet mass in units of Earth mass (M_⊕_). Blue circles and squares mark out the estimated pressure‐temperature conditions at CMB in the solar giant planets (Saturn, Uranus, and Neptune), according to the work by Guillot and Nettelmann et al., [39, 40] respectively.

To evaluate the planetary implications of these superionic behaviors, we superimposed the core‐mantle boundary (CMB) conditions of diverse planetary bodies onto the calculated pressure–temperature dynamical‐state diagrams (Figure 4). The calculated dynamical‐state regions overlap with P–T conditions estimated for several deep planetary interiors of solar giant planets (Saturn, Uranus, Neptune) as well as extrasolar super‐Earths [38, 39, 40]. Such overlap indicates that these compounds could exhibit these dynamical states if they are thermodynamically stable under the corresponding conditions, rather than demonstrating their chemical stability or abundance. For terrestrial and ocean‐type exoplanets ranging from 1 to 9 Earth masses (M_⊕_), the estimated CMB conditions intersect different dynamical regimes of AlOH and AlH_3_O_4_. AlOH generally remains within or close to the solid regime under these conditions, whereas AlH_3_O_4_ can access superionic or liquid‐like regimes over a broader range of planetary P–T conditions due to its lower superionic‐transition temperature. Similarly, the estimated deep‐interior conditions of Uranus, Neptune, and Saturn fall within the superionic or liquid regimes of these compounds. Nevertheless, their actual abundance additionally depends on bulk composition, redox state, volatile chemical potentials, and competition with other mineral and fluid phases. This limitation is particularly important for ice giants, where the abundance and distribution of Al at the relevant depths remain poorly constrained.

The liquid‐like diffusion of protons in the superionic states could contribute to substantial protonic transport. We estimated the corresponding conductivity of superionic AlOH and AlH_3_O_4_ using the Nernst–Einstein relation (Figure S16). At 400 GPa and 7000 K, AlOH yields a Nernst–Einstein conductivity estimate of approximately 3.3 S cm^−1^, while AlH_3_O_4_ reaches approximately 14 S cm^−1^ at 330 GPa and 3000 K. The larger value of AlH_3_O_4_ primarily reflects its higher proton concentration and greater proton mobility. We note that the Nernst–Einstein relation neglects correlations among mobile protons, and the reported values should therefore be regarded as approximate estimates rather than exact ionic conductivities. These values are comparable to or higher than the reported conductivity of highly conductive post‐perovskite (>10^2^ S m^−1^) but remain several orders of magnitude below those of Fe‐rich metallic core (∼10^5^–10^6^ S m^−^^1^) [41, 42]. Therefore, if these phases are present in sufficient abundance in planetary interiors, their protonic transport could contribute to electrically conductive regions.

We further examined several possible reactions involving common deep‐Earth materials: (1) AlOOH + FeO_2_ + 2H_2_ → AlOH + FeO + 2H_2_O; (2) AlOOH + FeH + 1/2O_2_ → AlOH + FeOOH; (3) AlOOH + FeO + H_2_O + O_2_ → AlH_3_O_4_ + FeO_2_. The calculated reaction energetics show that reactions (1) and (2) become favorable relative to the specified reactants at approximately 86 GPa, whereas reaction (3) becomes favorable at approximately 27 GPa (Figure S17). These crossover pressures characterize only the energetics of the specified reactions and do not represent the thermodynamic stability limits or formation depths of AlOH and AlH_3_O_4_, which require comparison against all competing assemblages. Therefore, these reactions should be regarded only as possible chemical pathways under appropriate local compositional and redox conditions [43].

Importantly, the unconventional stoichiometries and superionic behaviors identified in the Al─O─H system may have implications for chemical and transport processes under extreme planetary conditions. The protonic transport associated with these superionic states could contribute to the local electrical conductivity of planetary interiors if such phases are present in sufficient abundance. In the superionic states, the dynamical decoupling between mobile protons and the relatively rigid Al–O framework could facilitate proton transport and influence local chemical redistribution over geological timescales. More broadly, these results highlight the possible chemical interplay between refractory Al‐bearing components and planetary volatiles such as H and O, consistent with recent predictions of silica–water/hydrogen and MgO–water compounds under extreme planetary conditions [44, 45].

Moreover, the discovery of AlH_3_O_4_ has broader prospects for inorganic and materials chemistry. It expands the frontiers of high‐pressure crystal chemistry, showing that extreme pressure can stabilize unconventional stoichiometries with distinct site‐dependent electronic and bonding environments, combining high protonic capacity with superionic conductivity. Such materials provide fresh insights for designing proton‐conducting solid electrolytes under extreme pressure, enriching our understanding of chemical behaviors in dense matter. Details of the computational methods are provided in the Supporting Information [46, 47, 48, 49, 50, 51, 52, 53, 54, 55, 56].

## 3 Conclusion

In summary, our exploration of high‐pressure Al─O─H chemical landscape reveals fascinating chemical behaviors beyond conventional stoichiometries. We identify *Pnma*‐AlOH and *P‐1*‐AlH_3_O_4_ as stable compositions on the ternary convex hull at 300 GPa. Notably, AlH_3_O_4_ exhibits pronounced site‐dependent charge differentiation between inequivalent oxygen sites under high pressure. Furthermore, AIMD simulations reveal superionic states in both compounds with increasing temperatures, where protons diffuse like a liquid within the solid Al–O framework, giving rise to potentially substantial protonic transport. The calculated dynamical‐state regimes overlap with pressure–temperature conditions expected in planetary interiors, indicating that such behavior could become relevant if these phases are thermodynamically present. This work not only expands the frontiers of high‐pressure crystal chemistry but also reshapes our understanding of chemical behaviors under extreme conditions.

## Author Contributions

**Zifan Wang**: formal analysis, validation, investigation, visualization, writing – review and editing, writing – original draft. **Ji Hoon Shim**: formal analysis, validation, resources, writing – review and editing. **Duck Young Kim**: conceptualization, supervision, formal analysis, validation, investigation, funding acquisition, project administration, writing – review and editing.

## Conflicts of Interest

The authors declare no conflicts of interest.

## Supporting information




**Supporting File 1**: advs78561‐sup‐0001‐SuppMat.docx.

**Supporting File 2**: advs78561‐sup‐0002‐SuppMat.zip.

## Data Availability

The data that supports the findings of this study are available in the supplementary material of this article.
