# Supplementary material for: Superionic Al─O─H Compounds With Unconventional Stoichiometries Under Extreme Planetary Conditions
Source: Adv Sci (Weinh). 2026 Nov 10:e78561. Online ahead of print. doi: 10.1002/advs.78561 (PMC13749108; doi:10.1002/advs.78561)
Supplement: Supplementary file 1 [file ADVS-9999-e78561-s001.docx]

Supporting Information

Superionic Al-O-H compounds with unconventional stoichiometries under extreme planetary conditions

Zifan Wang^[a,b]^, Ji Hoon Shim^[b]^, and Duck Young Kim*^[a]^

[a]	Dr. Zifan Wang, Prof. Duck Young Kim
Center for High Pressure Science & Technology Advanced Research (HPSTAR)
Shanghai 201203, P.R. China
E-mail: duckyoung.kim@hpstar.ac.cn

[b]	Dr. Zifan Wang, Prof. Ji Hoon Shim
Department of chemistry
Pohang University of Science and Technology
Pohang 37673, Republic of Korea

**Methods**

***Ab initio* calculations**

*Ab initio* calculations are performed using Vienna *ab initio* simulation package (VASP)^[46]^ with the projector augmented wave (PAW) method.^[47]^ We used the generalized gradient approximation (GGA) of Perdew-Burke-Ernzerhof (PBE)^[48]^ for the exchange-correlation functional with the cut-off energy of 1000 eV. We treat 3s^2^3p^1^, 2s^2^2p^4^ and 1s^1^ electrons as valence electrons for Al, O and H atoms, respectively. Monkhorst-Pack *k*-point mesh^[49]^ with 0.03∙(2π/Å) and 0.04∙(2π/Å) mesh resolution was respectively used for the structural optimization and phonon calculations. Phonon dispersions, ZPE and Raman spectra were calculated by using the density functional perturbation theory (DFPT) as encoded in VASP to calculate the force constants in real space and PHONOPY code.^[50]^ The Bader charge analysis is done by using the near-grid method to obtain the Bader surface without lattice bias. Structure predictions were performed using AIRSS. ^[51,52]^ The searches systematically explored Al*ₓ*O*_y_*H*_z_* compositions with *x*, *y*, and *z* independently ranging from 1 to 10. Simulation cells containing up to 30 atoms were included in the searches, and more than 40,000 candidate structures were generated in total. Random structures were generated with minimum interatomic-distance constraints to avoid unphysically short atomic contacts, without imposing predefined structural motifs.

**Molecular dynamics**

*Ab initio* molecular dynamics (AIMD) simulations were conducted in the NVT ensemble using the Langevin thermostat implemented in VASP. ^[53]^ A plane-wave cutoff energy of 800 eV and Γ-point sampling were employed, with a timestep of 0.5 fs and a total simulation time of 10 ps. The simulations employed 96-atom supercells for AlOH (32 Al, 32 O, and 32 H atoms) and 128-atom supercells for AlH_3_O_4_ (16 Al, 64 O, and 48 H atoms). To assess the effects of simulation time and system size on proton diffusion, additional 30 ps AIMD simulations were performed for representative superionic states.

Large-scale machine-learning molecular dynamics (MLMD) simulations were further carried out for 200 ps using 1152-atom and 1024-atom cells for AlOH and AlH_3_O_4_, respectively. We used the Deep Potential for Molecular Dynamics (DeePMD)^[54,55]^ to train the force potentials, in which a deep neural network is trained to learn and generate potentials using atomic coordinates, energies, and forces from AIMD simulations data. Separate potentials were trained for each P–T condition. We used 8000 configurations for training and 2000 configurations for testing. Several key training parameters are as follows: We choose se_e2_a as a descriptor which is constructed by full relative coordinates, the cut-off radius is 6.0, the number of neurons in each hidden layers of the embedding net are 10, 20, 40 and the fitting net are 100, 100, 100, the desired learning rate is 3.5×10^-8^ and the total training step is 10^6^. MLMD simulations were carried out using the Large-scale Atomic/Molecular Massively Parallel Simulator (LAMMPS)^[56]^ code with the force field potentials generated by DeePMD, employing periodic boundary conditions and a time step of 0.5 fs. The machine-learning potentials were validated against DFT energies and atomic forces (Fig. S11, S12).

The dynamical states were identified from the atomic MSDs together with structural information from radial distribution functions (RDFs). A state was classified as superionic when the H diffusion coefficient satisfied $D_{H}\geq1\times{10}^{-10}$m² s⁻¹, the MSD of H exhibited sustained long-time growth, and the Al and O sublattices remained non-diffusive. A liquid state was assigned when all atomic species exhibited long-range diffusion together with substantial broadening and loss of longer-range correlations in the RDFs. Temperatures were generally sampled at intervals of 1000 K and at intervals of 500 K near the dynamical crossovers. The crossover temperature was taken as the midpoint between two adjacent simulations assigned to different dynamical states, corresponding to a typical uncertainty of approximately ±250 K for the 500 K sampling interval. Each temperature was simulated independently starting from the corresponding relaxed crystalline structure rather than through sequential heating.

**Electrical conductivity**

The protonic conductivity was estimated using the Nernst–Einstein relation,

$$\sigma_{NE}= \frac{DNq^{2}}{k_{B}T}$$

where *D* is the proton self-diffusion coefficient, *N* is the number density of mobile H atoms, *T* is the temperature, and *q*=+*e* is the nominal charge assigned to a mobile proton in the Nernst–Einstein estimate. The proton diffusion coefficient *D* was obtained from the linear slope of the H mean-squared displacement (MSD) over 1–10 ps according to *D* = (1/6)d⟨r²⟩/d*t*. For the original AIMD dataset, D was obtained from linear fits over 1–10 ps. For the extended validation simulations, fitting windows of 5–30 ps for AIMD and 20–200 ps for MLMD were used. Statistical uncertainties for the extended trajectories were estimated by non-overlapping block analysis and are reported as standard errors of the mean. The carrier number density was calculated as *N* = *N*_H_/*V*, where *N*_H_ is the number of H atoms and *V* is the simulation-cell volume. All H atoms were treated as mobile carriers in the superionic state. The numerical values of *N*, *D*, and σ_NE_ used in the calculations are provided in Table S2. This nominal transport charge should not be confused with the Bader charge, which is obtained from a partitioning of the static electron density and is used here only as a descriptor of local chemical bonding. The Nernst–Einstein relation neglects correlations among mobile protons. Therefore, the reported σ_NE_ values should be regarded as approximate conductivity estimates. Correlation effects may be described by a Haven ratio, H_R_, such that the collective ionic conductivity σ = H_R_σ_NE_ may differ from the Nernst–Einstein value.

**Fig. S1** Convex hull of formation enthalpy on Al-O-H system at 300 GPa. The energetically stable structures lying on the convex hull are represented by blue circles. The metastable structures are represented by blue squares.

**Fig. S2** Convex hull of formation enthalpy on Al-O-H system at 100 GPa. The energetically stable structures lying on the convex hull are represented by blue circles.

**Fig. S3** Ternary convex-hull diagram of the Al–O–H system at 300 GPa, including both stable and metastable compositions obtained from the structure searches. The energy above the convex hull, ΔE_hull_, is represented by the symbol size, while the color scale denotes the formation enthalpy per atom.

**Fig. S4** Static reaction enthalpy of the direct deoxygenation pathway AlOOH → AlOH + 1/2O_2_ as a function of pressure.

**Fig. S5** Phonon spectra of AlOH at 10, 100, 500 and 1000 GPa.

**Fig. S6** Phonon spectra of AlH_3_O_4_ at 0, 200, 500 and 1000 GPa.

**Fig. S7** Band structures of AlOH at 10, 100, 500 and 1000 GPa.

**Fig. S8** Band structures of AlH_3_O_4_ at 0, 200, 600 and 1000 GPa.

**Fig. S9** Electron localization function (ELF) maps of AlOH and AlH_3_O_4_ at 300 GPa. The ELF of AlOH is shown on the (010) plane, while representative sections through the two inequivalent oxygen environments in AlH_3_O_4_ are shown on the (1 −3 5) and (−1 1 1) planes. O_I_ and O_II_ denote the O1–O4 and O5–O8 oxygen groups, respectively. The color scale represents the ELF value from <0.01 to >0.96.

**Fig. S10** Pressure-dependent projected density of states (PDOS) of AlOH and AlH_3_O_4_. The PDOS of AlOH is shown at 100, 300, and 1000 GPa, including the H-1s, O-2p, and Al-s+p contributions. For AlH_3_O_4_, the PDOS is shown at 300, 600, and 1000 GPa, with the O-2p states resolved into the two inequivalent oxygen groups, O_I_ (O1–O4) and O_II_ (O5–O8). The PDOS is averaged per atom within each group, and the Fermi level is set to zero.

**Fig. S11** The atomic energy (a) and the forces (b-d) of AlOH calculated at around 400 GPa, 7000 K using the machine-learning force field potential compared with the results from DFT calculations. The last 1000 structures’ data were chosen to show in the figures. The specific RMSE value is indicated in the figure.

**Fig. S12** The atomic energy (a) and the forces (b-d) of AlH_3_O_4_ calculated at around 330 GPa, 3000 K using the machine-learning force field potential compared with the results from DFT calculations. The last 1000 structures’ data were chosen to show in the figures. The specific RMSE value is indicated in the figure.

**Fig. S13** Convergence test of extended AIMD simulations for representative superionic states of AlOH and AlH_3_O_4_.

**Fig. S14** Convergence test of MLMD simulations for representative superionic states of AlOH and AlH_3_O_4_.

**Fig. S15** RDFs g(r) of AlOH and AlH_3_O_4_ in the solid, superionic and liquid state.

**Fig. S16** Nernst–Einstein estimates of protonic conductivity of AlOH or AlH_3_O_4_ at the corresponding pressure–temperature conditions.

**Fig. S17** Formation enthalpies of several possible routes producing AlOH or AlH_3_O_4_ involving several minerals abundant in Earth’s mantle as reactants.

**Fig. S18** Calculated Raman spectra of AlOH at 300 GPa.

| Space group | Lattice parameters (Å , °) | Atomic coordinate (fractional) |
| --- | --- | --- |
| AlOH: *Pnma* | a = 3.9894,  b = 2.2878,  c = 4.8348,  α = β = γ =90 | H 0.13075 0.25 0.0589  H 0.86925 0.75 0.9411  H 0.36925 0.75 0.5589  H 0.63075 0.25 0.4411  O 0.45545 0.75 0.17567  O 0.54455 0.25 0.82433  O 0.04455 0.25 0.67567  O 0.95545 0.75 0.32433  Al 0.24317 0.75 0.87508  Al 0.75682 0.25 0.12492  Al 0.25683 0.25 0.37508  Al 0.74318 0.75 0.62492 |
| AlH_3_O_4_: *P-1* | a = 5.4467,  b = 3.8702,  c = 6.68,  α = 145.92,  β = 133.1863,  γ = 63.3789 | H 0.58456 0.29438 0.60109  H 0.41544 0.70562 0.39891  H 0.92801 1.19398 0.93590  H 0.07199 -0.19398 0.06410  H 0.30258 0.61102 0.50132  H 0.69742 0.38898 0.49868  O 0.12474 1.29359 0.34628  O 0.87526 -0.29359 0.65372  O 0.52776 0.00231 0.30248  O 0.47224 0.99769 0.69752  O 0.93125 0.71276 0.20257  O 0.06875 0.28724 0.79743  O 0.66315 0.72542 0.94670  O 0.33685 0.27458 0.05330  Al 0.24908 0.50751 -0.24502  Al 0.75092 0.49249 1.24502 |

**Table. S1** Structural information of AlOH and AlH_3_O_4_ at 300 GPa.

AlOH

| P (GPa) | T (K) | N (m^-3^) | D_H_ (m^2^/s) | R^2^ | σ_NE_ (S/cm) |
| --- | --- | --- | --- | --- | --- |
| 100 | 4000 | 6.543 × 10^28^ | 4.023 × 10^-10^ | 0.934 | 0.12 |
| 100 | 5000 | 6.543 × 10^28^ | 7.665 × 10^-8^ | 0.964 | 18.65 |
| 100 | 6000 | 6.543 × 10^28^ | 1.336 × 10^-7^ | 0.988 | 27.09 |
| 300 | 6500 | 8.428 × 10^28^ | 6.135 × 10^-9^ | 0.982 | 1.48 |
| 300 | 7000 | 8.428 × 10^28^ | 1.470 × 10^-8^ | 0.988 | 3.29 |
| 300 | 7500 | 8.428 × 10^28^ | 7.991 × 10^-8^ | 0.985 | 16.70 |
| 300 | 8000 | 8.428 × 10^28^ | 1.802 × 10^-7^ | 0.995 | 35.30 |
| 300 | 9000 | 8.428 × 10^28^ | 2.091 × 10^-7^ | 0.988 | 36.40 |
| 300 | 10000 | 8.428 × 10^28^ | 1.865 × 10^-7^ | 0.982 | 29.22 |
| 500 | 7000 | 9.751 × 10^28^ | 9.421 × 10^-10^ | 0.904 | 0.24 |
| 500 | 8000 | 9.751 × 10^28^ | 4.580 × 10^-8^ | 0.974 | 10.38 |
| 500 | 9000 | 9.751 × 10^28^ | 4.115 × 10^-8^ | 0.970 | 8.29 |
| 700 | 9000 | 1.083 × 10^29^ | 2.164 × 10^-8^ | 0.958 | 4.84 |
| 700 | 10000 | 1.083 × 10^29^ | 9.431 × 10^-8^ | 0.983 | 18.98 |
| 900 | 9000 | 1.176 × 10^29^ | 3.923 × 10^-9^ | 0.984 | 0.95 |
| 900 | 10000 | 1.176 × 10^29^ | 1.374 × 10^-8^ | 0.965 | 3.00 |
| 900 | 11000 | 1.176 × 10^29^ | 4.389 × 10^-8^ | 0.991 | 8.72 |

AlH_3_O_4_

| P (GPa) | T (K) | N (m^-3^) | D_H_ (m^2^/s) | R^2^ | σ_NE_ (S/cm) |
| --- | --- | --- | --- | --- | --- |
| 100 | 3000 | 8.303 × 10^28^ | 2.499 × 10^-8^ | 0.986 | 12.86 |
| 100 | 4000 | 8.303 × 10^28^ | 1.225 × 10^-7^ | 0.994 | 47.29 |
| 100 | 5000 | 8.303 × 10^28^ | 2.321 × 10^-7^ | 0.995 | 71.66 |
| 300 | 2000 | 1.075 × 10^29^ | 1.180 × 10^-9^ | 0.944 | 1.18 |
| 300 | 3000 | 1.075 × 10^29^ | 2.094 × 10^-8^ | 0.987 | 13.95 |
| 300 | 4000 | 1.075 × 10^29^ | 5.111 × 10^-8^ | 0.965 | 25.53 |
| 300 | 5000 | 1.075 × 10^29^ | 1.902 × 10^-7^ | 0.994 | 76.02 |
| 500 | 3000 | 1.243 × 10^29^ | 3.025 × 10^-8^ | 0.989 | 23.29 |
| 500 | 4000 | 1.243 × 10^29^ | 7.820 × 10^-8^ | 0.992 | 45.17 |
| 500 | 5000 | 1.243 × 10^29^ | 1.482 × 10^-7^ | 0.991 | 68.47 |
| 500 | 7000 | 1.243 × 10^29^ | 2.322 × 10^-7^ | 0.994 | 76.65 |
| 700 | 2000 | 1.379 × 10^29^ | 4.225 × 10^-9^ | 0.983 | 5.42 |
| 700 | 3000 | 1.379 × 10^29^ | 3.262 × 10^-8^ | 0.995 | 27.88 |
| 700 | 4000 | 1.379 × 10^29^ | 6.512 × 10^-8^ | 0.983 | 41.74 |
| 700 | 5000 | 1.379 × 10^29^ | 1.271 × 10^-7^ | 0.998 | 65.16 |
| 700 | 8000 | 1.379 × 10^29^ | 1.961 × 10^-7^ | 0.982 | 62.84 |
| 900 | 2000 | 1.496 × 10^29^ | 6.690 × 10^-9^ | 0.963 | 9.30 |
| 900 | 3000 | 1.496 × 10^29^ | 3.571 × 10^-8^ | 0.993 | 33.11 |
| 900 | 4000 | 1.496 × 10^29^ | 6.587 × 10^-8^ | 0.997 | 45.81 |
| 900 | 5000 | 1.496 × 10^29^ | 1.391 × 10^-7^ | 0.993 | 77.41 |

**Table S2.** Proton diffusion coefficients and Nernst–Einstein conductivity estimates of AlOH and AlH_3_O_4_.
